# Bidirectional Relationships Between Loneliness, Social Isolation, and Physical Inactivity in the Household, Income and Labour Dynamics in Australia Cohort Study

**DOI:** 10.1093/abm/kaae043

**Published:** 2024-07-27

**Authors:** Ben J Smith, Michelle H Lim, Karine E Manera, Philayrath Phongsavan, Katherine B Owen

**Affiliations:** Prevention Research Collaboration, Sydney School of Public Health, Faculty of Medicine and Health, The University of Sydney, NSW 2006, Australia; Prevention Research Collaboration, Sydney School of Public Health, Faculty of Medicine and Health, The University of Sydney, NSW 2006, Australia; Prevention Research Collaboration, Sydney School of Public Health, Faculty of Medicine and Health, The University of Sydney, NSW 2006, Australia; Prevention Research Collaboration, Sydney School of Public Health, Faculty of Medicine and Health, The University of Sydney, NSW 2006, Australia; Prevention Research Collaboration, Sydney School of Public Health, Faculty of Medicine and Health, The University of Sydney, NSW 2006, Australia

**Keywords:** Physical activity, Loneliness, Social isolation, Cohort study

## Abstract

**Background:**

Cross-sectional studies show associations between loneliness, social isolation and physical inactivity. Cohort studies are shedding light on these relationships and further longitudinal investigations are needed.

**Purpose:**

This study aimed to assess the longitudinal and bidirectional associations between loneliness, social isolation, and physical inactivity.

**Methods:**

Data were drawn from five annual waves of the Household and Labour Dynamics of Australia Survey (2015–2019), providing a sample of 17,303 persons (mean age = 46.3 years [*SD* = 18.0], 49.4% female). Relationships between loneliness, social isolation, and physical inactivity were examined using cross-lagged panel modeling, with estimation of simultaneous cross-lagged effects across each wave. Models adjusted for sociodemographic factors, chronic disease status, psychological distress, and mutually for social isolation or loneliness. Moderation of associations by sex was explored.

**Results:**

There were modest lagged effects of physical inactivity on loneliness across the survey waves (odds ratio 1.16 [95% confidence interval 1.04–1.29] to 1.20 [1.07, 1.33]). A lagged effect of physical inactivity upon social isolation was only present across three of the waves (odds ratio 1.20 [1.02–1.41] to 1.23 [1.05–1.42]). While loneliness and social isolation showed lagged effects upon physical inactivity, these did not persist with adjustment for psychological distress.

**Conclusions:**

Longitudinal analysis found that physical inactivity consistently predicted loneliness, but not social isolation. After adjustment for confounding, loneliness and social isolation were not predictive of physical inactivity. While the strength of the associations was modest, further investigation is warranted of the type and dose of physical activity that is most beneficial for reducing loneliness.

## Teaser

Over five sequential years people who were physically inactive were more at risk of feeling lonely but not of becoming socially isolated. However, those who were lonely or isolated were not more likely to become physically inactive.

## Introduction

Driven by an expanding body of epidemiological evidence, and fueled by the widespread social disruptions caused by the coronavirus disease 2019 (COVID-19) pandemic, there is growing awareness about the significant personal and public health impacts of loneliness and social isolation [1–3]. While these conditions are often referred to interchangeably, they constitute distinct dimensions of social wellbeing: social isolation is having objectively few relationships of any kind, whereas loneliness is the subjective feeling of a lack of connection to others and a desire for more satisfying relationships [1]. Despite these differences, both loneliness and social isolation have been found to predict poor health outcomes, notably depression, cognitive decline in older people, coronary heart disease incidence and mortality, and premature death from all causes [4–6].

Efforts to understand and address loneliness and social isolation from a public health perspective have led to research about the demographic, psychological, and social factors with which they are related [7–9]. This has shed light on the life stages (e.g., youth, old age) and life events (e.g., separation, chronic illness) that are associated with an elevated risk of having poor social relationships, and emerging insights about the prevalence and correlates of episodic versus chronic loneliness and social isolation [10]. In terms of mitigating the harmful effects of loneliness and social isolation, there has been investigation of how these social conditions lead to biological (e.g., blood pressure, inflammation) and behavioral (e.g., smoking, alcohol intake) changes that represent targets for primary and secondary prevention strategies [11–13].

For researchers and practitioners concerned with chronic disease prevention and mental health promotion, the relationship between loneliness and/or social isolation and participation in physical activity (PA) is one in which there has been strong interest. Numerous cross-sectional studies have found that individuals experiencing loneliness or social isolation report significantly lower levels of PA. A survey of university students in the UK found that those who were more physically active were less likely to be classified as socially isolated [14]. A study of Brazilian high school students found that youth with lower participation in physical education classes and leisure time PA were more likely to report both social isolation and loneliness [15]. Studies among older people have suggested contextual variation and complexity in these relationships. Among Ghanaian older adults it was found that loneliness had a stronger association with PA among men than women, and in those reporting lower social participation [16]. In a study of German older adults those who were socially isolated reported lower amounts of outdoor PA, but not indoor PA [17].

These cross-sectional studies indicate a relationship between loneliness and social isolation and PA, but they do not confirm its directionality, and this has implications for how this evidence may be applied to public health policy and programs. For instance, the question of whether PA can lead to reduced loneliness and/or isolation over time is relevant to whether PA should be prescribed, not only for chronic disease prevention, but also for the improvement of social health. A systematic review of evidence about the relationship between PA and loneliness included three longitudinal studies in which PA was an exposure variable of interest [18]. In two of these, it was found to predict loneliness in adolescents, albeit among females only in one of the studies. More recently, the Irish Longitudinal Study of Ageing (ILSA) found that higher levels of walking (but not moderate- or vigorous-intensity PA) were associated with lower levels of loneliness over 3 years [19]. Over 16 years of follow-up in the United States Health and Retirement study weekly participation in moderate-intensity PA was associated with a 6% lower likelihood of future loneliness, however, increases in PA did not predict a deviation from a person’s current level of loneliness [20]. A number of intervention trials have tested the social wellbeing impacts of different types of PA, with a meta-analysis of 38 randomized controlled trials among older people finding that PA had a beneficial effect on social functioning, but inconclusive effects on loneliness and social isolation [21].

The opposite pathway of causality, from loneliness or isolation to PA, is one of interest to public health policymakers and practitioners engaged in tackling the pervasive problem of physical inactivity. Social support is the domain of social health that has been most consistently examined as a predictor of PA [22, 23], but this is a distinct component of relationships from loneliness and social isolation [24]. In the abovementioned systematic review of the relationship between PA and loneliness [18] there were four longitudinal studies included that examined loneliness as the prior exposure of interest. In all of the studies, loneliness was found to predict lower PA at the later measurement points. There are fewer studies that have explored whether social isolation is independently associated with PA participation over time. An analysis of data collected over 10 years in the English Longitudinal Study of Ageing found that social isolation at baseline predicted lower levels of moderate and vigorous PA over the follow-up period [25]. Notably, loneliness was not found to independently predict PA, which highlights the insights that may be gained from the examination of these exposures concurrently.

Despite the mixed findings about the relationship between PA and both social isolation and loneliness, theoretical models identify biological, psychological, and social mechanisms that may operate between these factors and provide a basis for hypothesizing that these relationships are bidirectional. The Loneliness Model of Hawkley and Cacioppo [11] posits that hypervigilance toward perceived social threats is a characteristic of loneliness that results in elevated stress, a diminished capacity to regulate thoughts and feelings, and lower self-control to enact behaviors such as PA. Considering how social isolation may influence PA, the extensive body of social network theory identifies multiple ways that the nature and strength of social ties can act to affect health behaviors, including the provision of social support, conferral of social norms, the psychological effects of relationships and attachments, and access to resources [26]. Individuals experiencing social isolation will have low exposure to positive network influences upon PA.

In regard to theoretical explanations for how PA may affect aspects of social health, the Conceptual Model of Loneliness developed by Lim et al. [10] states that loneliness is the result of the independent or combined effects of triggers (e.g., divorce, geographic relocation) and risk factors (e.g., poor physical or mental health). Physical inactivity can be positioned as playing a critical role in the emergence of health risk factors for loneliness, given its strong relationship with cardiovascular health, physical functioning, and mental wellbeing. A further perspective is offered by the coping styles model, described by Fokkema and Knipscheer [27], in which engagement in PA in group contexts may reduce loneliness by facilitating improved social networks and confidence, and more positive perceptions of relationships. The conceptual model of cohesion in sports teams [28], which has been applied in group-based exercise more broadly, offers insights into the role that PA can play in reducing social isolation. This identifies social cohesion as a critical dynamic within sports and exercise settings that is influenced by individual motivation and group integration, and when achieved, contributes to sustained group engagement.

On the basis of the available evidence and theory, it can be hypothesized that PA has reciprocal relationships with both loneliness and social isolation. Testing this proposition will require longitudinal designs where both loneliness and social isolation are measured as discrete constructs, with analytic methods that can assess bidirectional pathways of association. Where possible, it will be valuable to examine if these relationships are moderated by sex, given past studies that have shown that males and females have differential vulnerability to loneliness and social isolation [29, 30], and that the social environment has been found to have a significant association with PA among females [31, 32]. The Household, Income and Labour Dynamics in Australia (HILDA) Survey is a large panel study with annual waves of data collection that provides this opportunity. The purpose of the present study was to determine the direction and strength of the predictive relationships between loneliness, social isolation, and physical inactivity over multiple sequential waves of the HILDA Survey.

## Method

### Study Design

The HILDA Survey is a household panel study that commenced in 2001 with an enrollment of 13,969 individuals using a multistage, structured sampling methodology [33]. This entailed the probabilistic selection of 448 Census Collection Districts (CCD) from across Australia (each comprising 200–250 households), then between 24 and 32 dwellings within each CCD, and up to three households within each dwelling (with a total household response rate of 66%). In 2011, the sample was replenished through replication of the multistage sampling in a further 125 CCDs (with a household response rate of 69%), generating an annual number of survey participants of approximately 17,600. The present study includes data collected over five annual survey waves, from 2014 until 2019 (before the onset of the COVID-19 pandemic). The HILDA Survey received institutional ethics approval from the University of Melbourne Faculty of Business and Economics Human Ethics Advisory Committee (#1647030). Study methods and results are reported following the Strengthening the Reporting of Observational Studies in Epidemiology (STROBE) Statement for cohort studies [34].

### Study Participants

To be eligible for inclusion, HILDA Survey participants must be 15 years over and have resided, or be expected to reside, in Australia for 12 months or more. For the present study, an analytic sample was drawn of individuals who participated in the survey and provided complete data in all waves from 2015 until 2019.

### Measures

Data collection in the HILDA Survey is conducted using a combination of interviewer-administered and self-complete methods. The survey includes measures of employment, education, income and expenditure, relationship status and satisfaction, time use, life events, height and weight, health status, quality of life, health behaviors, social interaction and support, financial stress, psychological distress, and additional topics on selected years [33]. In order to improve the accuracy of the collected data, and to reduce the risk of social desirability bias, the demographic, employment, and economic information is gathered by the interviewer-administered survey, whereas more personal and sensitive information (e.g., health behaviors, psychological distress) is obtained by the self-completed questionnaire.

The exposure and outcome variables examined in this study were loneliness, social isolation, and physical inactivity. Loneliness was measured with three items in which respondents were required to rate their agreement on a 7-point Likert scale, namely: “People don’t come to visit me as often as I would like,” “I often need help from other people but can’t get it,” and “I often feel very lonely.” The same 7-point Likert response scale was used for measuring social isolation, for which there were four items: “There is someone who can always cheer me up when I’m down,” “I enjoy the time I spend with the people who are important to me,” “When something’s on my mind, just talking with the people I know can make me feel better,” and “When I need someone to help me out, I can usually find someone.” These three and four-item scales have been each found to have internal reliability and construct validity [35], with psychometric analysis showing that an appropriate cut-point for the classification of loneliness is a median item score less than 4, and for social isolation a median item score of less than 4. PA was measured by a single question in which respondents were required to indicate how many times per week they usually participated in moderate or more intensive PA for at least 30 min. Those reporting up to 1–2 occasions per week were classified as physically inactive.

Demographic and health status measures were included as covariates in this study. The demographic covariates, drawn from the 2015 survey wave, were: age in years (15–29, 30–44, 45–59, 60–74, or 75 and over); sex (male, female); employment (full-time, part-time, retired, unemployed, or other); household income in Australian dollars (less than $80,000, $80,000–149,999, or $150,000 and over); country of birth (Australia, other English-speaking country, or other non-English-speaking country); and marital status (married/defacto, never married, divorced/separated, or widowed). Health status measures obtained from 2015, 2017, and 2019 survey waves were chronic disease status (Yes if reporting at least one long-term condition, or No), and psychological distress measured by the Kessler 10 scale (high or very high [scores 22–50] or low to moderate [scores 10–21]) [36].

### Data Analysis

**Fig. 1.**
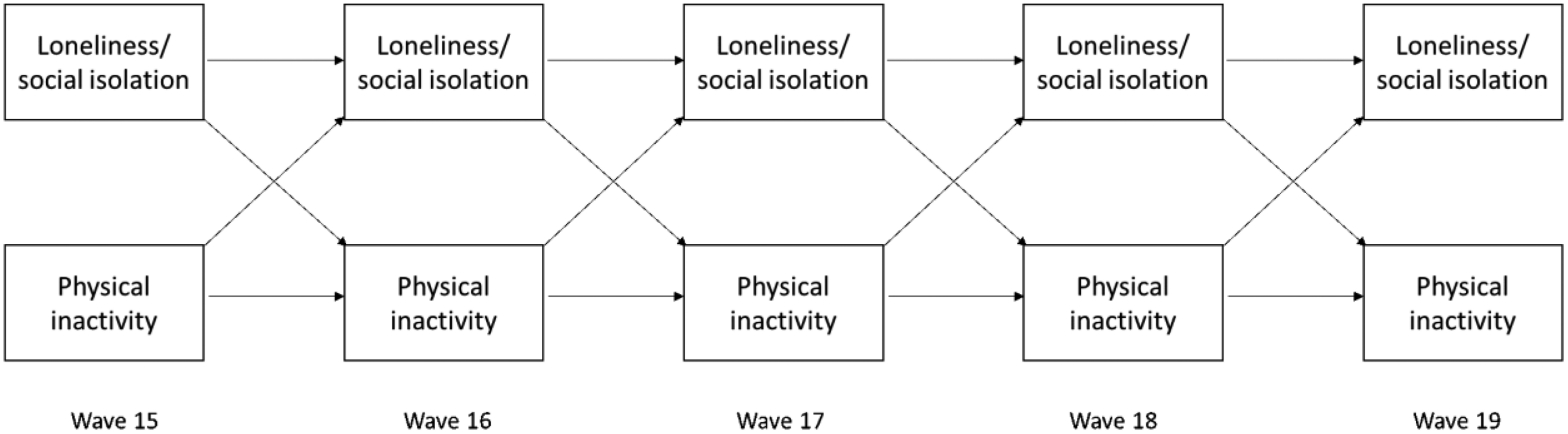
Structure for testing of bidirectional associations between loneliness, social isolation, and physical inactivity across five waves of the HILDA Survey. *Note*: Diagonal arrows represent cross-lagged effects and horizontal arrows represent autoregressive effects. *HILDA* Household, Income and Labour Dynamics in Australia.

We conducted a path analysis of a cross-lagged panel model to examine the longitudinal bidirectional relationships between loneliness or social isolation and physical inactivity (Fig. 1). We estimated the cross-lagged effects (i.e., the impact of one variable at a previous wave on the current values of another) of physical inactivity on loneliness or social isolation, and loneliness or social isolation on physical inactivity simultaneously. In the first model (Model 1), we included the outcome (i.e., loneliness or social isolation) from the previous wave. Model 2 adjusted for two non-time-varying covariates, sex and country of birth, and time-varying covariates including age, employment, income, marital status, and chronic conditions. Model 3 additionally adjusted for psychological distress (time-varying). The final model with loneliness as the outcome also adjusted for the time-varying covariate, social isolation; the final model with social isolation as the outcome adjusted for the time-varying covariate, loneliness (Model 4). The cross-lagged panel modelling (CLPM) accounted for missing and incomplete data using maximum likelihood estimation. To assess model fit, we calculated goodness-of-fit statistics including the Bayesian information criterion and Akaike information criterion. Additional exploratory analysis of the cross-lagged relationships between loneliness, social isolation, and physical inactivity stratified by sex was conducted (results shown in Supplementary Materials 1–4. All analyses were conducted using Stata/BE 17.0 (StataCorp LLC) statistical software.

## Results

There were 17,303 HILDA participants who completed surveys in all waves from 2015 to 2019. Table 1 shows that there was an even distribution of participants in the 15–29, 30–44, and 45–59 years age groups, accounting for about 70% of the sample, with the remainder aged 60 years and over. Approximately half were female, and around four in five were born in Australia or another English-speaking country. While the majority were working full-time or part-time, approximately one in six were retired. Just over half were in the lowest bracket of household income in 2015 (<$80,000), and this proportion declined incrementally across the waves. One-in-three participants were single, while three in five reported being in a couple relationship. Around one-quarter of participants had a chronic health condition, and the fraction reporting high or very high psychological distress was about one-in-six. In regard to the exposure variables that are the focus of this analysis, there was stability across Waves 15–19 in the proportions who were classified as physically inactive (42.9%–45.7%), lonely (12.4%–13.5%), and socially isolated (4.8%–5.7%).

**Table 1:** Demographic Characteristics of Participants Across Waves 15–19 of the HILDA Survey

	Wave 15		Wave 16		Wave 17		Wave 18		Wave 19	
	*N*	%	*N*	%	*N*	%	*N*	%	*N*	%
All persons	17,303	100	17,303	100	17,303	100	17,303	100	17,303	100
Age—mean yrs (*SD*)	46.3 (18.0)		47.3 (18.0)		48.3 (18.0)		49.3 (18.0)		50.3 (18.0)	
Age groups (yrs)										
15–29	4,075	23.6	4,191	24.2	4,105	23.7	4,012	23.2	3,543	20.5
30–44	3,733	21.6	3,877	22.4	3,915	22.6	3,917	22.6	3,877	22.4
45–59	3,861	22.3	3,971	23.0	3,935	22.7	3,870	22.4	3,745	21.6
60–74	2,841	16.4	2,962	17.1	3,027	17.5	3,093	17.9	3,125	18.1
75+	1,053	6.1	1,197	6.9	1,277	7.4	1,316	7.6	1,367	7.9
Missing	1,740	10.1	1,105	6.4	1,044	6.0	1,095	6.3	1,646	9.5
Sex										
Male	7,603	43.9	7,812	45.2	7,744	44.8	7,627	44.1	7,341	42.4
Female	8,541	49.4	8,767	50.7	8,723	50.4	8,581	49.6	8,318	48.1
Missing	1,159	6.7	724	4.2	836	4.8	1,095	6.3	1,644	9.5
Country of birth										
Australia	12,253	70.8	12,804	74.0	12,842	74.2	12,831	74.2	12,395	71.6
English speaking	1,464	8.5	1,516	8.8	1,491	8.6	1,452	8.4	1,384	8.0
Other	1,761	10.2	1,830	10.6	1,816	10.5	1,803	10.4	1,749	10.1
Missing	1,825	10.6	1,153	6.7	1,154	6.7	1,217	7.0	1,775	10.3
Employment status										
Employed F/T	6,465	37.4	6,726	38.9	6,872	39.7	6,913	40.0	6,730	38.9
Employed P/T	3,255	18.8	3,358	19.4	3,350	19.4	3,426	19.8	3,213	18.6
Unemployed	539	3.1	633	3.7	518	3.0	517	3.0	513	3.0
Retired	2,971	17.2	3,169	18.3	3,295	19.0	3,319	19.2	3,376	19.5
Other	2,305	13.3	2,295	13.3	2,202	12.7	2,021	11.7	1,805	10.4
Missing	1,768	10.2	1,122	6.5	1,066	6.2	1,107	6.4	1,666	9.6
Income (AUD)										
<$80,000	8,903	51.5	8,978	51.9	8,751	50.6	8,278	47.8	7,848	45.4
$80–149,000	3,417	19.8	3,596	20.8	3,452	20.0	3,508	20.3	3,341	19.3
$150,000+	3,525	20.4	3,678	21.3	3,896	22.5	4,149	24.0	4,140	23.9
Missing	1,458	8.4	1,051	6.1	1,204	7.0	1,368	7.9	1,974	11.4
Marital status										
Single	9,902	57.2	10,258	59.3	10,341	59.8	10,264	59.3	10,030	58.0
Part of a couple	5,579	32.2	5,894	34.1	5,812	33.6	5,820	33.6	5,500	31.8
Missing	1,822	10.5	1,151	6.7	1,150	6.6	1,219	7.0	1,773	10.2
Health condition										
Yes	4,380	25.3	4,468	25.8	4,908	28.4	4,715	27.3	4,634	26.8
No	11,099	64.1	11,686	67.5	11,240	65.0	11,369	65.7	10,896	63.0
Missing	1,824	10.5	1,149	6.6	1,155	6.7	1,219	7.1	1,773	10.3
Psychological distress										
Low	8,970	51.8			9,371	54.2			8,783	50.8
Moderate	3,021	17.5			3,368	19.5			3,223	18.6
High	1,568	9.1			1,823	10.5			1,770	10.2
Very high	882	5.1			1,044	6.0			1,155	6.7
Missing	2,862	16.5			1,697	9.8			2,372	13.7
Physical activity										
Inactive	7,415	42.9	8,138	47.0	7,905	45.7	7,864	45.5	7,651	44.2
Active	7,023	40.6	7,560	43.7	7,626	44.1	7,538	43.6	7,265	42.0
Missing	2,865	16.6	1,605	9.3	1,772	10.2	1,901	11.0	2,387	13.8
Lonely										
No	12,283	71.0	13,452	77.7	13,192	76.2	13,033	75.3	12,590	72.8
Yes	2,149	12.4	2,240	13.0	2,304	13.3	2,336	13.5	2,316	13.4
Missing	2,871	16.6	1,611	9.3	1,807	10.4	1,934	11.2	2,397	13.9
Socially isolated										
No	13,589	78.5	14,817	85.6	14,506	83.8	14,447	83.5	14,003	80.9
Yes	838	4.8	870	5.0	985	5.7	924	5.3	905	5.2
Missing	2,876	16.6	1,616	9.3	1,812	10.5	1,932	11.2	2,395	13.8

*HILDA* Household, Income and Labour Dynamics in Australia.

### Bidirectional Associations Between Loneliness and PA

The unadjusted analysis of the lagged effects of loneliness upon physical inactivity (Model 1) found that those who reported being lonely in Waves 15–18 of the survey had higher odds of being classified as inactive in each subsequent wave (Table 2). Across the four waves, the unadjusted odds ratios (ORs) ranged from 1.27 to 1.39. After adjustment for demographic characteristics and chronic disease status (Model 2), the lagged effects of loneliness upon physical inactivity were attenuated, but remained significant at each wave (ORs 1.16–1.27). However, with further adjustment for psychological distress (Model 3), the lagged effect of loneliness only remained significant at Wave 17 (OR 1.19, 95% confidence interval [CI] 1.06–1.34). Likewise, when social isolation was added as a covariate (Model 4), it was only at Wave 17 that a significant lagged effect of loneliness upon physical inactivity was detected (OR 1.18, 95% CI 1.05–1.32).

**Table 2:** Logistic Regression Coefficients for the CLPM Investigating the Bidirectional Lagged Effects of Loneliness and Physical Inactivity

	Model 1 OR (95% CI)	Model 2 OR (95% CI)	Model 3 OR (95% CI)	Model 4 OR (95% CI)
Physical inactivity in the next wave				
Loneliness at				
Wave 15	1.36 (1.22, 1.51)	1.22 (1.09, 1.37)	1.10 (0.97, 1.23)	1.09 (0.96, 1.22)
Wave 16	1.29 (1.16, 1.44)	1.18 (1.06, 1.32)	1.03 (0.91, 1.15)	1.02 (0.91, 1.14)
Wave 17	1.39 (1.25, 1.55)	1.27 (1.13, 1.41)	1.19 (1.06, 1.34)	1.18 (1.05, 1.32)
Wave 18	1.27 (1.14, 1.42)	1.16 (1.04, 1.29)	1.02 (0.91, 1.14)	1.01 (0.90, 1.14)
Loneliness at the next wave				
Physical inactivity at				
Wave 15	1.35 (1.22, 1.50)	1.27 (1.14, 1.42)	1.17 (1.04, 1.30)	1.16 (1.04, 1.29)
Wave 16	1.33 (1.20 (1.47)	1.22 (1.10, 1.36)	1.11 (1.00, 1.24)	1.10 (0.99, 1.23)
Wave 17	1.41 (1.28, 1.56)	1.31 (1.18, 1.45)	1.19 (1.07, 1.32)	1.20 (1.08, 1.33)
Wave 18	1.42 (1.29, 1.58)	1.32 (1.19, 1.47)	1.20 (1.07, 1.33)	1.20 (1.07, 1.33)

*Note*: Model 1 adjusted for loneliness or physical inactivity in the previous wave when either of these variables was the outcome. Model 2 additionally adjusted for age, sex, employment, income, country of birth, marital status, and chronic conditions. Model 3 additionally adjusted for psychological distress. Model 4 additionally adjusted for social isolation.

Model fit indices: Model 1—AIC = 82,880.8, BIC = 83,057.0; Model 2—AIC = 81,830.48, BIC = 83,343.16; Model 3—AIC = 80,292.62, BIC = 82,040.29; Model 4—AIC = 80,111.16, BIC = 81,917.57.

*AIC* Akaike information criterion; *BIC* Bayesian information criterion; *CI* confidence interval; *OR* odds ratio.

Examination of the ORs for the lagged effects of loneliness upon physical inactivity stratified by sex showed stronger and more consistent relationships among females (see Supplementary Material 1). Notably, the ORs generated in Model 3 for females at Waves 17 (OR 1.19, 95% CI 1.00–1.43) and 18 (OR 1.22, 95% CI 1.02–1.45) and in Model 4 at Wave 18 (OR 1.21, 95% CI 1.02–1.44) were significant, but this was not the case among males.

Analysis of the lagged effects of physical inactivity upon loneliness also showed statistically significant unadjusted associations at each wave, with ORs in the range of 1.33–1.42 (Table 2). While slightly attenuated, these effects remained after adjustment for demographic factors and chronic disease status, with ORs ranging from 1.22 to 1.32. By contrast with findings for the predictive association between loneliness upon physical inactivity, after further adjustment for psychological distress, there was still statistically significant lagged effects of physical inactivity upon loneliness in all waves, with ORs of 1.11–1.20. In three waves (15, 17, 18), these lagged effects remained stable after adjustment for social isolation. Stratification of these models by sex did not reveal consistent differences between females and males (see Supplementary Material 2).

### Bidirectional Association Between Social Isolation and PA

The unadjusted analysis found that the lagged effects of social isolation upon physical inactivity were statistically significant from Waves 15 to 17 (ORs 1.28–1.37), but not at Wave 18 (Table 3). The lagged effects at Waves 15–17 remained statistically significant after adjustment for demographic factors and chronic disease status, with ORs ranging from 1.19 to 1.31. However, with further adjustment for psychological distress, it was only at Wave 17 that a significant predictive association was found between social isolation upon physical inactivity (OR 1.25, 95% CI 1.06–1.47), and this remained stable after adjustment for loneliness (OR 1.23 95% CI 1.04–1.45). Stratification of the models by sex did not reveal consistently stronger relationships among either females or males (see Supplementary Material 3).

**Table 3:** Logistic Regression Coefficients for the CLPM Investigating the Bidirectional Lagged Effects of Social Isolation and Physical Inactivity

	Model 1 OR (95% CI)	Model 2 OR (95% CI)	Model 3 OR (95% CI)	Model 4 OR (95% CI)
Physical inactivity in the next wave				
Social isolation at				
Wave 15	1.32 (1.09, 1.52)	1.24 (1.05, 1.46)	1.13 (0.95, 1.34)	1.10 (0.93, 1.31)
Wave 16	1.28 (1.09, 1.52)	1.19 (1.01, 1.41)	1.05 (0.89, 1.25)	1.06 (0.90, 1.27)
Wave 17	1.37 (1.17, 1.60)	1.31 (1.12, 1.54)	1.25 (1.06, 1.47)	1.23 (1.04, 1.45)
Wave 18	1.15 (0.98, 1.35)	1.08 (0.92, 1.28)	0.97 (0.82, 1.14)	0.95 (0.80, 1.12)
Social isolation at the next wave				
Physical inactivity at				
Wave 15	1.45 (1.24, 1.70)	1.35 (1.15, 1.58)	1.22 (1.04, 1.43)	1.20 (1.02, 1.41)
Wave 16	1.44 (1.25, 1.66)	1.39 (1.20, 1.61)	1.24 (1.07, 1.44)	1.23 (1.05, 1.42)
Wave 17	1.07 (0.92, 1.24)	1.00 (0.86, 1.17)	0.89 (0.77, 1.04)	0.87 (0.75, 1.02)
Wave 18	1.12 (0.97, 1.29)	1.05 (0.91, 1.22)	0.92 (0.79, 1.06)	0.89 (0.76, 1.03)

*Note*: Model 1 adjusted for social isolation or physical inactivity when each of these variables was the outcome. Model 2 additionally adjusted for age, sex, employment, income, country of birth, marital status, and chronic conditions. Model 3 additionally adjusted for psychological distress. Model 4 additionally adjusted for loneliness.

Model fit indices: Model 1—AIC = 68,878.95, BIC = 69,055.19; Model 2—AIC = 68,107.35, BIC = 69,620.03; Model 3—AIC = 67,227.63, BIC = 68,916.54; Model 4—AIC = 66,994.95, BIC = 68,742.61.

*AIC* Akaike information criterion; *BIC* Bayesian information criterion; *CI* confidence interval; *OR* odds ratio.

Analysis of the lagged effects of physical inactivity upon social isolation revealed inconsistent associations across the waves. As shown in Table 3, at Waves 15 and 16 there were statistically significant lagged effects of physical inactivity, with higher odds of social isolation in all models. With full adjustment for demographic factors, chronic disease status, psychological distress, and loneliness (Model 4), the OR for the effects of physical inactivity upon social isolation were 1.20 (95% CI 1.02–1.41) in Wave 15 and 1.23 (95% CI 1.05–1.42) in Wave 16. In contrast, there were no statistically significant lagged effects of physical inactivity upon social isolation detected in any of the models in Waves 17 and 18. There were not found to be marked differences between females and males when the models were stratified by sex (see Supplementary Material 4).

## Discussion

This study has found limited evidence of bidirectional associations between measures of social connectedness and physical inactivity over time. The most consistent relationship, across all five waves of measurement, was physical inactivity predicting higher levels of loneliness in the subsequent year. However, the observed ORs were below the minimum size considered to represent a practically important effect [37], indicating that alternative methods of analysis should be considered to examine the scale and distributional variation of the relationships between these variables (e.g., quantile regression). Physical inactivity had a far less consistent relationship with social isolation, indicating that the objective presence of social relationships is less affected by PA participation than subjective satisfaction with those connections. This highlights the importance of measuring these constructs of loneliness and social isolation independently in social health research.

This study builds on recent longitudinal investigations which have also shown that PA predicts lower levels of loneliness, although with variations in findings that likely reflect differences in the measurement and analysis of these variables. In the ILSA, continuous minutes of walking in the past 7 days were found to have a negative relationship with scores on the five-item loneliness measure over 3 years [19]. Analysis of the US Health and Retirement cohort over 16 years found that at least one occasion of moderate-intensity sports or PA (of unspecified duration) in a usual week predicted a lower likelihood of being classified as lonely on a single-item measure [20]. In the present study, those undertaking more than 1-2 occasions of moderate- or vigorous-intensity PA in a usual week, of at least 30 min duration, were consistently less likely to be classified as lonely in the next year. Together, these studies suggest that small increments of PA can be beneficial for reducing loneliness. The mechanisms at work in this relationship may be physiological, through the effects of PA on hormonal activity and mood states [38, 39] and/or psychological, through the relationship between PA participation and self-esteem [40, 41], either of which may increase sociability and positivity and lower the risks of loneliness. It remains unclear what type (individual vs. group based) and dose (duration and frequency) of PA are most likely to activate mechanisms that will result in reductions in loneliness that are of clinical and public health significance.

Contrary to what was found concerning its consistent relationship with loneliness, physical inactivity was only found to predict social isolation in 2 years of the CLPM analysis. Surprisingly, it was found across all models in those years, but not in any of the models in the latter 2 years. The fact that the relationship was found in models that included adjustment for age in Waves 15 and 16 would indicate that the maturation of the cohort is unlikely to be the factor causing this change. We are not aware of any societal factors that could have overridden the effect of physical inactivity upon social isolation in Waves 17 and 18, as might have been the case if measures were collected in 2020–2021 (when COVID lockdowns were implemented). The lack of repeated associations across all waves of measurement may in part be because the PA measure did not differentiate between activity conducted individually and that which is undertaken in groups (often described as organized PA). A benefit that is widely reported from organized PA, that includes sports and group-based exercise in leisure facilities and other venues, is that it provides an opportunity for building relationships and improving social inclusion [42, 43].

A selection of longitudinal studies have reported that loneliness and/or social isolation predict physical inactivity, although these findings have also been variable. One of the first studies to examine this relationship prospectively was a small longitudinal study of middle-aged and older adults in the USA, which found that loneliness was associated with lower odds of PA participation over 2 years, and an increased likelihood of decline toward inactivity [44]. In the abovementioned ILSA, social isolation, but not loneliness was predictive of lower moderate and vigorous PA over 3 years [19]. A cohort study of Canadian adults found that social isolation had a bivariate association with lower PA over 5 years, however after adjusting for confounders, it was the characteristics of social networks (i.e., levels of trust, presence of other exercisers) that were significant predictors of PA [45].

While our study found that loneliness and social isolation predicted physical inactivity in models that included demographic factors and chronic disease status as covariates (in Model 2), these relationships did not persist when there was further adjustment for psychological distress (in Model 3). The exception to this was among females, where in two of the waves (17 and 18), there was a small, but significant predictive relationship between loneliness and physical inactivity after psychological distress was taken into account, which may reflect a stronger influence of social connection and social support upon PA participation by females. Notwithstanding this, the lack of consistency in the findings about these relationships and the attenuation of the ORs between Models 2 and 3, indicate that poor mental health largely accounts for the predictive relationship between lack of social connection and physical inactivity.

Strengths of this study included its large and diverse national sample of adults, facilitating generalizability to the Australian population. The administration of annual measurements to HILDA Survey participants enabled the testing of lagged effects of the exposures of interest over four sequential years. In addition, the measures of loneliness and social isolation that were used have been found to have good reliability and validity in psychometric analysis. It should be noted, however, that the items used to measure social isolation referred only to the functional qualities of social relationships (reflecting a lack of “social connectedness”), and not the objective frequency of social contacts as commonly assessed in other measures of this construct. In this study, PA was measured using a single-item question which, despite having similarity to other validated PA measures [46], does not have known measurement properties. Furthermore, as noted above, the PA measure did not enable calculation of the time spent in different types of activity (e.g., organized vs. non-organized), that may have different relationships with loneliness and social isolation. The limitations of the CLPM method that was used should be acknowledged, including that this analysis strategy does not examine within-person variance and it assumes the rank-order stability of constructs drops to zero in the long term (which is not the case for most individual constructs). However, we considered this approach to be suitable given that neither the assessment of individual differences, nor the stability of measured constructs, were objectives of this study.

## Conclusion

Our study indicates that promoting PA may be beneficial for reducing loneliness. However, given that the relationship between physical inactivity and loneliness was of modest strength, more research is needed to understand what type and how much PA should be undertaken for it to have an impact on loneliness that is of clinical and public health significance. This further evidence would provide guidance for primary care and mental health practitioners in regard to the appropriate recommendations for the prevention of loneliness among patients. The evidence concerning the opposite path of causality, from loneliness and social isolation to PA was inconsistent, suggesting that the subjective and objective characteristics of social connections are not strong predictors of PA. This examination of longitudinal and bidirectional relationships between aspects of social wellbeing and lifestyle behaviors provides evidence to unlock how the frequently reported associations between these determinants of health can be applied in policy and programs.

## Supplementary Material

Supplementary material is available at *Annals of Behavioral Medicine* online.

kaae043_suppl_Supplementary_Material

## Data Availability

The data used in these analyses are available on request to the Melbourne Institute: Applied Economic and Social Research at the University of Melbourne which is the manager and data custodian for the HILDA study (https://melbourneinstitute.unimelb.edu.au/hilda).
