# Supplementary material for: Bidirectional Relationships Between Loneliness, Social Isolation, and Physical Inactivity in the Household, Income and Labour Dynamics in Australia Cohort Study
Source: Ann Behav Med. 2024 Jul 27;58(9):619–27. doi: 10.1093/abm/kaae043 (PMC11305128; doi:10.1093/abm/kaae043)
Supplement: kaae043_suppl_Supplementary_Material [file kaae043_suppl_supplementary_material.docx]

**Electronic supplementary material**

1. Logistic regression coefficients for the CLPM investigating the bi-directional lagged relationship between loneliness and physical inactivity, stratified by sex

|  | Model 1  OR (95% CI) | Model 2  OR (95% CI) | Model 3  OR (95% CI) | Model 4  OR (95% CI) |
| --- | --- | --- | --- | --- |
|  | Physical inactivity in the next wave | | | |
| Loneliness at: |  |  |  |  |
| *Females* |  |  |  |  |
| Wave 15 | **1.40 (1.18, 1.65)** | **1.25 (1.06, 1.48)** | 1.13 (0.95, 1.36) | 1.13 (0.94, 1.35) |
| Wave 16 | 1.15 (0.97, 1.36) | 1.07 (0.91, 1.27) | 0.95 (0.8, 1.14) | 0.94 (0.79, 1.13) |
| Wave 17 | **1.43 (1.21, 1.68)** | **1.31 (1.11, 1.55)** | **1.19 (1.00, 1.43)** | 1.18 (0.99, 1.41) |
| Wave 18 | **1.47 (1.24, 1.74)** | **1.36 (1.14, 1.61)** | **1.22 (1.02, 1.45)** | **1.21 (1.02, 1.45)** |
| *Males* |  |  |  |  |
| Wave 15 | **1.26 (1.04, 1.52)** | 1.16 (0.96, 1.41) | 1.04 (0.84, 1.28) | 1.03 (0.84, 1.27) |
| Wave 16 | **1.42 (1.18, 1.72)** | **1.34 (1.10, 1.62)** | 1.17 (0.95, 1.42) | 1.16 (0.94, 1.41) |
| Wave 17 | 1.16 (0.97, 1.4) | 1.07 (0.89, 1.29) | 1.01 (0.83, 1.24) | 1.00 (0.82, 1.22) |
| Wave 18 | 1.18 (0.98, 1.42) | 1.06 (0.87, 1.28) | 0.9 (0.74, 1.09) | 0.90 (0.74, 1.1) |

2. Logistic regression coefficients for the CLPM investigating the bi-directional lagged relationship between physical inactivity and loneliness, stratified by sex

|  | Model 1  OR (95% CI) | Model 2  OR (95% CI) | Model 3  OR (95% CI) | Model 4  OR (95% CI) |
| --- | --- | --- | --- | --- |
|  | Loneliness at the next wave | | | |
| Physical inactivity at: |  |  |  |  |
| *Females* |  |  |  |  |
| Wave 15 | **1.37 (1.17, 1.61)** | **1.31 (1.11, 1.54)** | **1.19 (1, 1.4)** | **1.18 (1.00, 1.40)** |
| Wave 16 | **1.37 (1.17, 1.60)** | **1.27 (1.08, 1.5)** | 1.17 (0.99, 1.38) | 1.15 (0.97, 1.36) |
| Wave 17 | **1.49 (1.27, 1.74)** | **1.41 (1.2, 1.65)** | **1.29 (1.1, 1.52)** | **1.30 (1.11, 1.53)** |
| Wave 18 | **1.39 (1.20, 1.63)** | **1.3 (1.11, 1.52)** | 1.17 (0.99, 1.37) | 1.16 (0.99, 1.37) |
| *Males* |  |  |  |  |
| Wave 15 | **1.42 (1.18, 1.69)** | **1.37 (1.14, 1.64)** | **1.26 (1.05, 1.52)** | **1.25 (1.04, 1.51)** |
| Wave 16 | **1.3 (1.09, 1.54)** | 1.18 (0.99, 1.42) | 1.06 (0.87, 1.27) | 1.04 (0.86, 1.26) |
| Wave 17 | **1.28 (1.08, 1.52)** | 1.19 (0.99, 1.41) | 1.07 (0.89, 1.28) | 1.08 (0.90, 1.29) |
| Wave 18 | **1.45 (1.22, 1.73)** | **1.35 (1.12, 1.61)** | 1.2 (0.99, 1.44) | **1.21 (1.00, 1.45)** |

3. Logistic regression coefficients for the CLPM investigating the lagged relationship between social isolation and physical inactivity, stratified by sex

|  | Model 1  OR (95% CI) | Model 2  OR (95% CI) | Model 3  OR (95% CI) | Model 4  OR (95% CI) |
| --- | --- | --- | --- | --- |
|  | Physical inactivity in the next wave | | | |
| Social isolation at: |  |  |  |  |
| *Females* |  |  |  |  |
| Wave 15 | 1.10 (0.84, 1.45) | 1.03 (0.78, 1.36) | 0.93 (0.7, 1.23) | 0.90 (0.68, 1.20) |
| Wave 16 | **1.46 (1.11, 1.91)** | **1.33 (1.01, 1.75)** | 1.22 (0.93, 1.62) | 1.23 (0.93, 1.63) |
| Wave 17 | **1.50 (1.14, 1.97)** | **1.43 (1.09, 1.88)** | 1.31 (0.99, 1.73) | 1.26 (0.95, 1.68) |
| Wave 18 | 1.22 (0.92, 1.63) | 1.13 (0.85, 1.52) | 1.01 (0.75, 1.36) | 0.99 (0.73, 1.33) |
| *Males* |  |  |  |  |
| Wave 15 | **1.49 (1.14, 1.94)** | **1.40 (1.07, 1.84)** | 1.29 (0.98, 1.7) | 1.25 (0.95, 1.65) |
| Wave 16 | 1.07 (0.81, 1.42) | 1.01 (0.76, 1.35) | 0.86 (0.64, 1.16) | 0.87 (0.65, 1.17) |
| Wave 17 | **1.35 (1.05, 1.72)** | **1.28 (1.00, 1.64)** | 1.25 (0.97, 1.62) | 1.21 (0.94, 1.57) |
| Wave 18 | 1.14 (0.88, 1.47) | 1.03 (0.79, 1.34) | 0.88 (0.67, 1.15) | 0.88 (0.67, 1.15) |

4. Logistic regression coefficients for the CLPM investigating the bi-directional lagged relationship between physical inactivity and social isolation, stratified by sex

|  | Model 1  OR (95% CI) | Model 2  OR (95% CI) | Model 3  OR (95% CI) | Model 4  OR (95% CI) |
| --- | --- | --- | --- | --- |
|  | Social isolation at the next wave | | | |
| Physical inactivity at: |  |  |  |  |
| *Females* |  |  |  |  |
| Wave 15 | **1.40 (1.09, 1.79)** | 1.28 (0.99, 1.65) | 1.2 (0.93, 1.55) | 1.16 (0.90, 1.51) |
| Wave 16 | **1.49 (1.17, 1.91)** | **1.41 (1.10, 1.81)** | 1.24 (0.96, 1.61) | 1.19 (0.91, 1.54) |
| Wave 17 | 1.08 (0.84, 1.39) | 1.00 (0.77, 1.30) | 0.91 (0.70, 1.19) | 0.87 (0.66, 1.13) |
| Wave 18 | **1.28 (1.00, 1.64)** | 1.17 (0.91, 1.51) | 1.00 (0.77, 1.30) | 0.96 (0.74, 1.25) |
| *Males* |  |  |  |  |
| Wave 15 | **1.55 (1.20, 2.01)** | **1.47 (1.13, 1.92)** | 1.28 (0.98, 1.68) | 1.26 (0.96, 1.65) |
| Wave 16 | **1.40 (1.12, 1.75)** | **1.33 (1.06, 1.67)** | 1.21 (0.96, 1.54) | 1.21 (0.96, 1.53) |
| Wave 17 | 1.04 (0.83, 1.32) | 0.95 (0.75, 1.21) | 0.85 (0.66, 1.08) | 0.84 (0.66, 1.08) |
| Wave 18 | 1.11 (0.88, 1.38) | 1.03 (0.82, 1.3) | 0.91 (0.72, 1.15) | 0.89 (0.70, 1.12) |
